# Delusion and Delirium in Neurodegenerative Disorders: An Overlooked Relationship?

**DOI:** 10.3389/fpsyt.2021.808724

**Published:** 2022-01-18

**Authors:** Daniele Urso, Valentina Gnoni, Marco Filardi, Giancarlo Logroscino

**Affiliations:** ^1^Department of Clinical Research in Neurology, Center for Neurodegenerative Diseases and the Aging Brain, Pia Fondazione Cardinale G. Panico, University of Bari Aldo Moro, Bari, Italy; ^2^Department of Neurosciences, Institute of Psychiatry, Psychology and Neuroscience, King's College London, London, United Kingdom; ^3^Department of Basic Medicine, Neuroscience, and Sense Organs, University of Bari Aldo Moro, Bari, Italy

**Keywords:** delirium, delusion, neurodegeneration, Alzheimer's disease, Dementia with Lewy bodies, frontotemporal dementia, neurodegenerative disease

## Abstract

Delusions are part of the neuropsychiatric symptoms that patients suffering from neurodegenerative conditions frequently develop at some point of the disease course and are associated with an increased risk of cognitive and functional decline. Delirium is a syndrome characterized by acute onset of deficits in attention, awareness, and cognition that fluctuate in severity over a short time period. Delusions and delirium are frequently observed in the context of neurodegeneration, and their presence can easily mislead clinicians toward a misdiagnosis of psychiatric disorder further delaying the proper treatment. Risk factors for developing delusion and delirium in neurodegenerative conditions have been investigated separately while the possible interplay between these two conditions has not been explored so far. With this study, we aim to achieve a more comprehensive picture of the relationship between delusions and delirium in neurodegeneration by analyzing prevalence and subtypes of delusions in different neurodegenerative disorders; providing an overview of clinical tools to assess delusions in neurodegenerative patients and how delusions are covered by delirium assessment tools and discussing the possible common pathophysiology mechanisms between delusion and delirium in neurodegenerative patients. A more extensive characterization of the relationship between delusions and delirium may help to understand whether delusions may constitute a risk factor for delirium and may ameliorate the management of both conditions in patients with neurodegenerative disorders.

## Introduction

The term delusion is widely used in scientific literature, however, its conceptualization is challenging and delusions remain among the most elusive concepts in psychiatry (1). The current diagnostic and statistical manual of mental disorders (DSM-V) defines delusions as fixed false beliefs that are not amenable to change in light of conflicting evidence (2). Delusions are considered a multidimensional phenomenon characterized along with a number of themes/delusional beliefs which may vary across the different psychiatric and neurological disorders (3). Delusions are frequently experienced by patients with neurodegenerative disorders during the course of their illness and (4), together with hallucinations, are symptoms that may mimic a psychiatric condition (psychosis mimics) and lead to a misdiagnosis between neurocognitive and psychiatric disorders (5). Moreover, delusions are part of the constellation of symptoms defining delirium, a syndrome characterized by acute onset of deficits in attention, awareness, and cognition that fluctuate in severity over a relatively short time span (typically days or weeks) (6, 7) and similarly may be easily mistaken for a psychiatric disorder. Delusions and delirium are frequently observed during the disease course of neurodegeneration (8, 9), and are associated with overall worse cognitive and functional outcomes (10, 11).

Notwithstanding the prevalence of delusions in patients with neurodegenerative disorders during episodes of delirium has been scarcely investigated, likely due to the fact that when assessing delirium neuropsychiatric symptoms, including delusions and hallucinations, are typically clustered together.

An early study by Lerner et al. that investigated delirium in a small cohort of AD patients showed that a history of hallucinations and paranoid delusions were more common in patients delirium (12). More recently a handful of studies have shown higher frequency and severity of delusions in patients with dementia and delirium compared with patients with dementia without delirium (13). The objective of this article is to achieve a more comprehensive picture of the possible interplay between delusions and delirium in patients with neurodegenerative disorders. To achieve this goal we firstly analyse the prevalence and subtypes of delusional beliefs in neurodegenerative disorders of different etiology (namely, Alzheimer's Disease, Parkinson's Disease, Dementia with Lewy bodies and Frontotemporal lobar degeneration); then, we provide an overview of clinical tools and rating scales to assess delusions in neurodegenerative patients and how delusions are explored by the most commonly used delirium assessment tools. Finally, we discuss the possible common pathophysiology mechanisms between delusion and delirium in neurodegenerative patients.

## Delusions in Neurodegenerative Disorders

### Alzheimer's Disease

Alzheimer's Disease (AD) is the most prevalent neurodegenerative disorder accounting for an estimated 60-80% of all cases of dementia (14). The underlying mechanisms and causes for AD are still not completely understood. However, it has been established that histopathological characteristics of AD include extracellular β-amyloid peptides deposition and formation of neurofibrillary tangles arising from the intracellular accumulation of hyperphosphorylated Tau proteins (15–17). Clinically, AD presents as a slow progressive disease with a long preclinical phase and disease progression which spans several stages and includes preclinical Alzheimer's disease, mild cognitive impairment (MCI) due to Alzheimer's disease, and Alzheimer's disease dementia (18, 19). More recently, it has been advanced the concept of AD as a continuum, spanning from an asymptomatic phase where pathophysiological changes are already evident, to the symptomatic phase where biomarkers changes continue and symptoms progressively worsen leading to the eventual loss of independence (20). Neuropsychiatric symptoms (NPS) such as aggression, depression, apathy, and psychosis are increasingly recognized to be significant features of Alzheimer's disease and are reported to be associated with faster cognitive decline (21–23). The concept of mild behavioral impairment (MBI) has been recently proposed as a diagnostic construct aimed to identify patients with NPS, who have an increased risk of developing dementia, but may not present cognitive symptoms (24). Delusions in AD have been described since Alois Alzheimer's index case of A. Deter, who presented initially with emotional distress and delusions of infidelity, followed by cognitive impairment (25).

In early observational studies, the prevalence of delusions in AD was reported to be between 10 and 73 % (26). Differences in assessment tools used along with the different definitions of delusions and the clinical population investigated may be responsible for the wide difference in prevalence across studies. A systematic review and meta-analysis that investigated the prevalence of neuropsychiatric symptoms in AD, reported an overall pooled delusions prevalence of 31% although with considerable heterogeneity across individual studies results (27). Indeed the authors highlighted that the prevalence was influenced by population origin and disease duration. Similarly, a previous study showed that a longer duration of illness was independently associated with the presence of psychosis and delusions (28), although this finding was not been confirmed in subsequent studies (29). By contrast, it has been shown that the severity of cognitive decline is predictive of delusions. The cumulative incidence rates for delusions in patients with probable AD in a study by Paulsen et al. were 20.1, 36.1, 49.5, and 51.3%, at 1-, 2-, 3-, and 4-year postbaseline evaluations, respectively. Another study by D'Onofrio et al. found that patients with AD who had delusions had higher grades of cognitive impairment and a more severe stage of dementia assessed by the Clinical Dementia Rating Scale (CDR) (30). Delusions have been associated with increased risk of cognitive and functional decline (31), and increased risk of mortality (30) although additional studies are required to confirm this association. Delusions appear to be consistently associated with older age whilst contrasting evidence are reported regarding sex, other demographic variables as well as the use of medications and medical comorbidities as possible risk factors for developing delusions (32, 33). Delusions are frequently associated with other psychological conditions (34–36) like depression and in turn, may increase the risk of developing other neuropsychiatric symptoms including agitation aggression, depressed mood, apathy, irritability, aberrant motor activity, sleep disturbances, eating disorders and hallucinations (30). Most notably, up to 44% of patients with AD may experience delusions associated with hallucinations. D'Onofrio et al. found that delusions of theft were the main delusion's subtype in patients with AD (50.4%) whilst delusions of abandonment, persecution and infidelity were found in 25.6, 15.7, and 8.3% of cases, respectively (30). Misidentification phenomena have also been reported in AD including phantom boarder delusion and the “one's house is not the one's home” delusion (37, 38). More rarely AD patients exhibit paranoid delusions. A study by Naasan et al. found paranoid delusions were reported in 9.1 % of autopsy-confirmed patients with AD, persecutory in 1.8 % and misidentification of people in 4.5 % (39).

### Dementia With Lewy Bodies and Parkinson's Disease

Dementia with Lewy bodies (DLB) is the second most common degenerative dementia. It is characterized by visual hallucinations, cognitive fluctuation, parkinsonism and dementia. Parkinson's disease dementia (PDD) is defined as dementia that arises in the context of established Parkinson's disease (PD), and shares both clinical and neuropathological characteristics with DLB (40). It is believed that DLB and PDD represent a spectrum disorder with similar cortical and subcortical Lewy body pathology but different onset of cognitive and motor symptoms. The landscape of psychosis in Parkinson's disease (PD) has been redefined in 2007 by consensus recommendations from an international workgroup (41). In PD, psychosis may exist on a continuum in which minor phenomena progress from hallucinations with retained insight to hallucinations with loss of insight and delusions (42). Delusions in PD are less frequent than hallucinations. Prevalence estimates range from 3 to 16% (43–46) and are higher in PD dementia (PDD; 19–51%) (47–49). Delusions are most commonly paranoid and the most common theme is infidelity (44). Delusional misidentification syndromes, a specific subset of delusions characterized by pathological familiarity, including Capgras syndrome and reduplicative paramnesia have also been described in PD (50–52). By contrast, delusions are very common in DLB, occurring in up to 60 % of cases (53, 54), and have become one of the supportive features for the clinical diagnosis of DLB (55, 56). A study by Nagahama et al. showed that hallucinations, misidentification experiences and delusions were found, respectively, in 78, 56, and 25% of DLB cases (57). In particular, misidentifications of people (17%), of objects (28%), reduplication of people (10%) and phantom boarder (11%) were the most frequent misidentification symptoms whilst delusions of theft (14%) and persecution (11%) were the most common delusions. Furthermore, Naasan et al. have also shown that in patients with DLB and AD co-pathology, delusions were more frequent than in patients with AD and Frontotemporal lobar degeneration (FTLD) (39). In 20% of cases, they were characterized by more than one subtype and in 10% of cases, they occurred in the first 3 years of the disease.

In patients with DLB and comorbid AD pathology, the most frequent delusions were reported to be of paranoid content (19%) followed by misidentification of people (13.8%) and place (10.3%). The persecutory content and of grandeur occurred in 8.5% of cases and of jealousy in 5.2% (39).

Aarsland et al. investigated delusions in DLB, and PD with and without dementia. The authors found that delusions were significantly more common in DLB (57%) than PDD (29%) and PD without dementia (7%) (53). Phantom boarder phenomenon and paranoid ideation were the most common delusion in these disorders. Finally, it has been shown that patients with DLB who presented delusions have higher disease severity, more neuropsychiatric symptoms and are more cognitively impaired (58). Frequent and distressing delusions in DLB have been associated with higher caregiver burden and with worse patient's quality of life compared with patients with other neurodegenerative disorders (58).

### Frontotemporal Lobar Degeneration

Frontotemporal lobar degeneration (FTLD) is a clinically and pathologically heterogeneous syndrome associated with degeneration of the frontal and anterior temporal lobes and clinically characterized by a progressive impairment of behavior and/or language. Clinical variants include behavioral FTD, mainly characterized by changes in personality and behavior, and FTLD- associated aphasic syndromes, namely primary progressive aphasia (PPA) which is divided into semantic, progressive non-fluent and logopenic variants (59–61). The majority of pathologies associated with FTLD clinical syndromes include either tau-positive (FTLD-TAU) or TAR DNA-binding protein 43 (TDP-43)-positive (FTLD-TDP) inclusion bodies whilst AD pathology has been found in more than 70% of logopenic PPA cases (61).

A study estimated delusion prevalence of 14% in FTD patients, with paranoid and somatic being the most more frequent types of delusions (62). More recently, Sha et al. found that delusions were the presenting neuropsychiatric symptom in 21% of patients with bvFTD with an expansion in C9orf72, in 18% of patients with FTD and motor neuron disease (FTD-MND) carrying the C9orf72 mutation, and in 10.5% of the FTD-MND non-carrier (63). Naasan et al. investigated psychotic symptoms in autopsy confirmed neurodegenerative pathology and found that patients with FTLD-TDP were significantly more likely to have delusions (34.8%) compared to patients with AD and FTLD-tau (16.2 and 10.5%, respectively). In FTLD-TDP, delusions tend to occur in the first 3 years of the disease and are characterized by bizarre content in 7.4%, paranoid content in 26.5% and persecutory content in 14.5% of cases. Furthermore, patients with FTLD-TDP had also frequently delusions of misidentification and grandiosity and were primarily seen in patients with FTLD-TDP-B pathology at a frequency of 9.4%. By contrast, In FTLD-tau delusions were 8.3% of paranoid content and 3.8% persecutory (39).

## Delusions Assessment in Neurocognitive Disorders and Cognitive Impairment

Several assessment tools have been developed according to the DSM-V definition of delusions, mainly aimed at allowing clinicians to ascertain the presence and intensity of delusion in different clinical settings and to investigate the association between specific delusional beliefs and psychiatric disorders of different etiology. Nonetheless, instruments specifically developed to evaluate the presence and features of delusional beliefs in patients suffering neurocognitive disorders and mild cognitive impairment are lacking. Indeed, the general approach of studies on delusion in neurocognitive disorders consists in administering multidimensional scales that assess delusions within a wide range of behavioral disturbances. Therefore, information on sensitivity, specificity and accuracy of these tools in identifying the presence of delusions are not currently available.

The Neuropsychiatric Inventory (NPI) and the Behavioral Pathology in Alzheimer's Disease Rating Scale (BEHAVE-AD) have been the two most widely used instruments for this purpose followed by the Consortium to Establish a Registry for Alzheimer's Disease-Behavior Rating Scale for Dementia (CERAD-BRSD) (Table 1) (64). The NPI has been by far the most commonly used instrument (65). The NPI is based on a structured interview conducted with a caregiver and/or patient's relative that takes ~10-15 min. The interview has to be administered to a caregiver sufficiently good at assessing the patient's behavior and requires a trained psychologist or clinician. The NPI assesses 12 neuropsychiatric disturbances, including delusions, experienced by the patient over the past month *via* screening questions (66). Each neuropsychiatric disturbance is rated for presence (range 0-4) and severity (range 0-3). NPI total score ranges between 0 and 144 with higher scores indicating greater behavioral disturbance. A domain score ≥ 4 is indicative of clinically significant symptoms.

**Table 1:** Neuropsychiatric symptoms assessment tools covering the dimension of delusion.

**Delusion assessment instruments**	**Time to complete**	**Rater qualification**	**Neuropsychiatric symptoms investigated**	**Delusions investigated**	**Time period evaluated**
Neuropsychiatric inventory	10-15 min	Trained raters Clinical raters Nurses without specialized training [NPI-NH] Self-administered [NPI-Q]	- Delusions - Hallucinations - Depression - Anxiety - Apathy - Irritability - Elation - Disinhibition - Agitation - Aberrant motor - Behavior - Appetite and eating - Disturbance - Nighttime behavior disturbances	- Persecution - Theft - Spouse infidelity - Unwelcomed guests in the house - Abandonment - People are not who they claim to be - One's house is not one's home - Characters from television or magazines are real or in the room	Previous month
Behavioral pathology in Alzheimer's disease rating scale	20 min	No formal training required	- Paranoid and delusional ideation - Hallucinations - Activity disturbance - Aggressiveness - Diurnal rhythm disturbances - Affective disturbances - Anxieties and phobias	- People are stealing things - Place in which the patients is residing is not his/her home - Spouse or the caregiver is an imposter - Abandonment - Infidelity	Previous 2 weeks
Consortium to establish a registry for Alzheimer's disease-behavior rating scale for dementia	20–30 min	Trained raters Clinical raters	- Depressive features - Inertia - Psychotic features - Vegetative features - Irritability/aggression - Behavioural dysregulation	- Spouse is unfaithful - Being abandoned - Spouse is an imposter - TV characters are real - People are in house - Dead person is alive - One's home is not one's home	Previous month

If the delusions are present a scripted question is asked in order to assess their features and the caregiver is asked to rate delusions frequency, severity, and associated distress. More in detail the NPI assess the following delusional beliefs: delusions of persecution and theft (paranoid delusions), spouse infidelity (Othello syndrome), unwelcomed guests in the house (phantom boarder delusion), abandonment, people not being who they claim to be (Capgras syndrome), delusion that “one's house is not one's home” and delusion that characters from television or magazines are real or in the room.

The NPI exhibit excellent psychometric proprieties (67), has been translated into more than 40 languages (66), and is available in different versions including a version for unsupervised completion and a version for use in residential settings (68, 69).

The BEHAVE-AD is the second most commonly used instrument to assess delusional beliefs in AD and related dementia (70). The BEHAVE-AD is a clinical rating scale that evaluates changes in patient's behavior over the previous 2 weeks. It is based on an interview conducted with the caregiver or a patient's relative which takes ~20 min. The original version of the scale rated symptoms based on severity and provides separate measures for delusions and behavioral abnormalities. Each symptom is rated for presence (0-1) and severity (range 0-3), total BEHAVE-AD score ranges between 0 and 75.

Five delusional beliefs are investigated by the BEHAVE-AD: delusion that people are stealing things, delusion that the place in which the patient is residing is not his/her home, delusion that the spouse or the caregiver is an imposter, delusion of abandonment and delusion of infidelity. The BEHAVE-AD display good psychometric properties (71), has been revised to provide information on both severity and frequency and has been validated for telephone-based administration (72). Finally, the CERAD-BRSD is a rating scale developed to assess behavioral abnormalities in patients with cognitive impairment (73). The CERAD-BRSD is administered to the patient's caregiver or relative and requires a trained interviewer. The CERAD-BRSD consist of 48 items investigating presence, frequency and severity of specific behavioral abnormalities; total score ranges between 0 and 167. It assesses the following delusional beliefs with respect to their frequency over the past month: belief that spouse is unfaithful, belief of being abandoned, belief that spouse is an imposter, belief that TV characters are real, belief that people are in the house, belief that dead person is alive and belief that “one's home is not one's home.” The CERAD-BRSD exhibits excellent psychometric properties (74), and has been used as an outcome measure in numerous intervention studies (75), but is more time- and training-demanding for administration compared to the NPI and the BEHAVE-AD.

More recently the Etiological assessment of psychotic symptoms in dementia (EAPSID) has been proposed as a tool to evaluate delusions in dementia patients from an etiological perspective (76). EAPSID provides a functional analysis of delusional beliefs assessed through the NPI or the BEHAVE-AD by inquiring on type of delusion, content of delusion, context and frequency of occurrence, where does the delusion occur, reactions of others to the delusions as well as the patients' verbal expressions of the delusions. The EAPSID seems a promising tool for identifying guidance programs for caregivers/relatives to efficiently manage the delusional beliefs experienced by the patients with a potentially positive impact on quality of life (77).

## Delusions Assessment in Neurocognitive Disorders and Cognitive Impairment

Regrettably, the domain of delusion is often overlooked by the instruments used to rate the presence and severity of delirium. A recent review of literature on delirium assessment tools reported that only four of the eleven most commonly used delirium assessment tools cover the domain of delusion (78). More importantly among the three most commonly used delirium assessment tools, i.e., the Confusion Assessment Method, the Delirium Rating Scale and the Memorial Delirium Assessment Scale only the two letters investigate the presence of delusion (79). Moreover, due to the intrinsic features of delirium (acute onset and rapid symptoms fluctuation), these instruments were developed to assess the presence of delusions over a very short time span (from a day to a few hours before the acute onset) thus precluding the possibility of assessing whether the presence of delusion could represent a risk factor for delirium.

## Pathophysiology and Hypotheses

**Figure 1:**
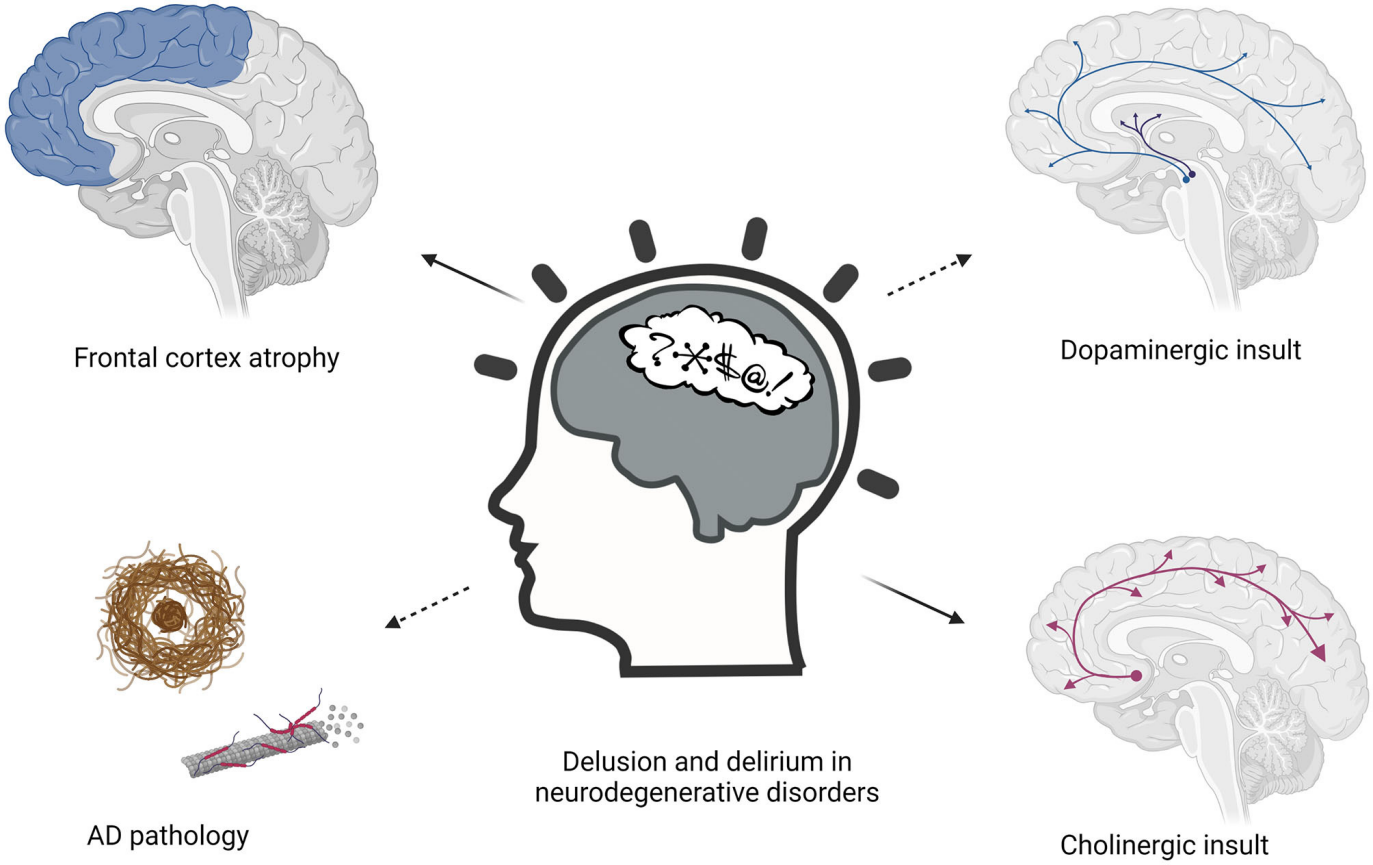
Pathophysiological hypothesis about the etiology of delusions and delirium in neurodegenerative disorders.

Various pathophysiological hypotheses have been proposed regarding the etiology of both delusions and delirium in neurodegenerative disorders (Figure 1). These hypotheses come from the observation that the development of these conditions is facilitated by the interplay of predisposing and precipitating factors (80, 81). Predisposing factors are older age, visual and hearing impairment, severe illness, cognitive impairment, depression, malnutrition, pressure ulcers, urinary incontinence, polypharmacy, which represent the baseline vulnerabilities of an older person (81, 82). Precipitating factors are the acute noxious insults or hospitalization-related factors that contribute to delirium, such as infection, metabolic imbalance, or surgery (83). Furthermore, different groups of drugs can trigger both delirium and delusions, particularly sedative-hypnotics with documented psychoactive effect, narcotics, and anticholinergic drugs (84, 85). Delirium could not be explained by a single etiological theorem. It is becoming increasingly clear that delirium may be the consequence of a neurotoxic event affecting a vulnerable brain, in the context of a neurotransmitter and inflammatory derangement.

Some evidence suggested that delusion can be the result of dysfunction of frontal lobe circuitry. A study by Zubenko et al. showed an association between psychosis and frontal lobe hypometabolism, as well as density of senile plaques and neurofibrillary tangles in the midfrontal cortex of AD patients (86). Another study by Paulsen et al. demonstrated that AD patients with psychotic symptoms will exhibit the typical cognitive deficits of AD but with particularly severe deficits in fronto-subcortical functions compared to patients without psychotic symptoms, confirming the so-called hypofrontality model (87). Similarly, other studies revealed a significant relationship between delusional thought and metabolic rates over frontal regions in both AD and DLB patients (88, 89). Muscarinic acetylcholine receptor density was increased in the frontal cortex of patients with AD presenting psychotic symptoms compared to AD without psychosis (90).

Recently, a new technique called atrophy network mapping identified a delusions network that included regions in the bilateral ventrolateral frontal, orbitofrontal frontal, and superior frontal cortices. Noteworthy Frontal lobe dysfunction has been associated also with delirium. Choi et al. investigated resting-state functional connectivity in patients with delirium (91). They found that dorsolateral prefrontal cortex activity and posterior cingulate cortex activity were inversely correlated in comparison subjects while patients experiencing an episode of delirium shows increased functional connectivity between the two regions. Another study demonstrated that longer duration of delirium was associated with smaller superior frontal lobe in Intensive Care Unit survivors (92). Therefore, data have shown that both delirium and delusions can be associated with changes in frontal cortex.

Among neurotransmitters, acetylcholine and dopamine, are the most frequently associated with delirium and delusions (93, 94). Many neurodegenerative conditions, including AD and DLB have been associated with an extensive loss of basal forebrain projection neurons, which is considered to be the major cholinergic output of the central nervous system (95, 96). Cholinergic basal forebrain atrophy occurs early in both diseases and predicts cognitive decline (97, 98). Also in PD, basal forebrain atrophy has been associated with early cognitive decline (99, 100). Dysregulation of the cholinergic system has been suspected in the pathophysiology of primary psychotic disorders (101, 102) and can contribute to behavioral disturbances in patients with dementia (103).

Interestingly, a study found that cholinergic basal forebrain volume is associated with future psychotic symptoms in PD patients (104). Moreover, serum anticholinergic activity (SAA) levels have been found to increase during the acute phase of delirium, have been linked with a higher burden of delirium symptoms (105), and decline with the resolution of delirium (106). On the other hand, the use of anticholinergic drugs is closely related to the occurrence of delirium (107).

A recent study on postoperative delirium found that the changes in the preoperative activity of Acetylcholinesterase (AChE), Butyrylcholinesterase (BuChE) and Choline acetyltransferase (ChAT) in CSF were associated with the development of postoperative delirium in elderly patients, which may be related to central cholinergic degradation (108). Although the cholinergic deficiency hypothesis suggests treatment with acetylcholinesterase inhibitors may prevent and improve delirium two double-blind, randomized trials failed to demonstrate a statistically significant effect of cholinesterase inhibitors in the prevention and treatment of postoperative delirium (109, 110).

Dopamine has also been involved in the pathophysiology of delirium. Ramirez-Bermudez et al. found that psychotic symptoms in delirious patients were related to increased CSF homovanillic acid (HVA) levels (111). Another study conducted by the same group confirmed that the association between delirium and CSF HVA concentration was significant also in patients without exposure to antipsychotics (112). The dopaminergic activation of the nigrostriatal pathway is critical for the production of hallucinations and perceptual changes (113), which can be present during delirium. Interestingly, psychotic symptoms have been associated with striatal denervation assessed with 123I-FP-CIT SPECT, in both PD and DLB patients (114, 115). Dopaminergic circuitry and dopaminergic medication have also a clear relationship with psychotic symptoms in PD and DLB (116) although that cannot fully explain the phenomenon. A hypothesis that has been raised is that denervation hypersensitivity of mesolimbic and mesocortical dopaminergic receptors predisposes patients to a hypersensitivity response which may manifest as psychosis (41). The link between the susceptibility to develop delirium/delusion and the dopaminergic disruption seen in synucleinopathies and in other neurodegenerative diseases could represent an important area for future research.

Kim et al. studied psychosis symptoms in patients with severe Alzheimer's disease who are cognitively normal. They showed that neuritic plaque severity, and not the NFTs, is associated with a higher risk of psychosis in this population (117). Similarly, reduced CSF amyloid Aß1-42 have been found in *de novo* PD patients who will develop psychosis (118), suggesting that delusions can be linked to amyloid pathology also in PD. Similarly, preclinical AD brain pathology has been recently linked to the occurrence of delirium in non-demented patients. Idland and colleagues observed a reduction in CSF A42, indicating amyloidosis, in hip fracture patients without dementia developing delirium (119).

The neuroinflammatory hypothesis of delirium, postulate that an acute peripheral event, such as infection or surgery, triggers a systemic inflammatory response and activation of microglia in the central nervous system (120). Peripheral inflammation can induce changes in CNS by several routes, including proinflammatory cytokines (121), endothelial activation with disruption of the blood-brain barrier (122), and microglial activation (123). Interleukin 6 (IL-6) has been strongly associated with the duration of delirium in non-demented patients (124). A study by Macdonald et al. found that the level of C-reactive protein, an acute-phase protein released in response to increasing concentrations of IL-6, predicted the onset and recovery of delirium (125). Neurodegenerative diseases have been also inextricably related to neuroinflammation (126). Persistent inflammatory stimulation is induced by endogenous, such as genetic mutation and protein aggregation, or environmental, such as infection, trauma, and drugs factors (6, 7). The inflammatory responses involve microglia and astrocytes and can lead to neurodegenerative diseases (127). Although neuroinflammation can be initially a protective response in the brain, persistent inflammatory responses are detrimental and may promote the onset and progression of neurodegenerative diseases (128). Field et al. hypothesize that decreased cholinergic function confers increased susceptibility to acute inflammation-induced cognitive deficits and found that cholinergic depletion predisposes to the development of acute cognitive deficits upon subsequent systemic inflammatory insult (129). This hypothesis may explain why patients with neurodegenerative disorders affecting the cholinergic basal forebrain, are particularly susceptible to developing delirium. While neuroinflammation has been linked with primary psychiatric disorders (130), it should be determined if it can have a role in the pathophysiology of psychiatric disorders related to neurodegeneration.

## Conclusions

Delusional beliefs and delirium are frequently observed during the course of dementia. Delusions and delirium when present in a neurodegenerative condition represent an additional disease burden significantly increasing the risk of institutionalization and posing relevant distress to the patients' caregiver and relatives. Furthermore, their characteristics can easily mislead clinicians toward a misdiagnosis of psychiatric disorder further delaying the proper treatment. The diagnosis of delirium may be even more challenging within the context of some types of neurodegenerative disorders, especially DLB and PD dementia, characterized by a “sensitivity” to minor insults, even subclinical, and recurrent complex visual hallucinations and delusions. There is not a clear indication so far on how to evaluate the presence of a pre-existing psychosis (including delusion) in patients with a neurocognitive disorder who develop delirium and the assessment or inclusion of pre-existing and long-established delusion is also not addressed by current standard tools. Therefore, the development of an instrument for accurate recognition of delusions in patients with neurodegenerative disorders who develop delirium is of the utmost importance. Delirium assessment instruments should consider the anticholinergic burden and should include a more extensive characterization of delusional beliefs specifically inquiring whether delusions are present in a time span (from week to months) typically not covered by the commonly used delirium rating scales. Such a tool may shed light on the relationship between delusions and delirium in neurodegeneration and may help to understand whether pre-existing delusion may constitute a risk factor for delirium and a prognostic factor for its clinical course. Further studies using multimodal neuroimaging are warranted to test the hypotheses of unbalance between different networks and neurotransmitter deficits in these conditions.

## Author Contributions

DU, VG, MF, and GL: conception and design of work and drafting the manuscript. All authors were involved in critical revision of the manuscript for important intellectual content.

## Conflict of Interest

The authors declare that the research was conducted in the absence of any commercial or financial relationships that could be construed as a potential conflict of interest.

## Publisher's Note

All claims expressed in this article are solely those of the authors and do not necessarily represent those of their affiliated organizations, or those of the publisher, the editors and the reviewers. Any product that may be evaluated in this article, or claim that may be made by its manufacturer, is not guaranteed or endorsed by the publisher.
